# A young boy with malignant hypertension and intracranial bleed

**DOI:** 10.4103/0971-4065.65308

**Published:** 2010-04

**Authors:** Manish Rathi, Sakhuja V, Munni Ray, Amanjeet Bal

**Affiliations:** **CPC Editor:** Assistant Professor of Nephrology; **CPC Chairperson:** Dean and Head of Nephrology; **Clinical Discussant:** Associate Professor of Pediatrics; **Pathology Discussant:** Assistant Professor of Histopathology

A 12-year-old male child presented to pediatric emergency with complaints of abnormal movements involving the right side of body, which rapidly progressed to alteration of sensorium along with repeated bouts of vomiting since last one day. He developed all problems acutely and was apparently well before this. In a local hospital he was noted to have weakness of right side of the body and was referred to PGI. There was no history of fever, loose stools, head trauma or excessive bleeding prior to this. The past history was totally unremarkable. The child was born to a gravida 2 mother by full-term normal vaginal delivery and there were no adverse prenatal events. His developmental milestones were normal. He was appropriately immunized.

He was born of a non-consanguineous marriage. His elder sibling had died at the age of 1.5 years due to some unknown illness. There was no history of contact with tuberculosis.

On examination, he weighed 25 kg, had no pallor, icterus, clubbing, edema or skin bleed. His heart rate was 140 beats/minute, respiratory rate 36 per minute, capillary filling time 2 seconds, blood pressure (BP) 220/160 mm Hg and oxygen saturation was 100% on room air. All the peripheral pulses were palpable. The BP in the upper limbs was 180/130 mm Hg and in the lower limbs was 170/120 mm Hg. Ophthalmoscopic examination showed bilateral disk blurring with hemorrhages.

On systemic examination, he had a Glasgow coma score of E2, M4, V2, both the pupils were 2 mm and reacted to light, and there was no cranial nerve palsy. There was hypotonia of all four limbs, more on the right side. The deep tendon reflexes were normally elicitable on the left side; but absent on the right side. Plantars were upgoing in both the legs. Neck rigidity and Kernig’s sign were positive. Cardiovascular, abdominal and respiratory examinations were normal. Investigations are shows in Tables 1 and 2.

**Table 1:** Investigations

	Date	
	26.08.06	28.08.06
Hb(g/dL)	16.9	14.4
TLC (/mm^3^)	11,000	10,100
DLC	N79 L16M4 E1	N72 L23M4 E1
Platelets	Adequate	Adequate
Blood film	N/N	N/N

	**26.08.06**	**30.08.06**

Prothrombintime	11”	10”
Prothrombintime index	91%	100%
PTTK	32”	26”

**Table 2:** Investigations (Contd.)

	Date											
	25/08	26/08	27/08	27/08	28/08	29/08	29/08	30/08	30/08	31/08	31/08
Sodium(mEq/L)	134	135	132	167	181	135	152	129	154	159	159
Potassium(mEq/L)	5.1	5.5	3.8	3.8	4.3	6.0	4.3	3.4	3.6	5.4	5.4
Urea(mg/dL)	35	35	32	79	79	61	79	59	73	320	80
Creatinine(mg/dL)	0.6	0.6	0.6	0.8	0.7	1.3	0.7	1.0	0.8	5.4	2.0
Bilirubin(mg/dL)							0.7		0.7		
TSP/Alb(g/dL)							6.9/3.6		5.7/2.7		
SGOT/SGPT(IL/L)					59/36		59/36				
Alkaline					136						
phosphatase(lU/L)											
Ca/P(mg/dL)					10.4/2.3		9.3				
CRP (mg/dL)							2.63				
Cholesterol(mg/dL)					219						
Bloodsugar (mg/dL)							116	487	326		247

Chest X-ray (26/08/06): Normal study; Abdominal ultrasound ( 28/08/06): Small left kidney (6.7 cm) with 1 cm calculus seen at the lower pole; Right kidney: 10.0 cm; NCCT head (25/08/06): Massive left basal ganglia bleed with midline shift and hydrocephalus; MRI brain (25/08/06): Massive left basal ganglia bleed with midline shift and hydrocephalus; NCCT head (30/08/06): Gross cerebral edema with infarct, no fresh bleed

Blood culture (26/08/06): sterile; Urine RE (28/08/06): protein, nil; sugar, nil; no RBC. Pus cells: 2–3/hpf. Oxalate crystals ++; Urine sugar (30/08/06): ++++; Urine electrolytes (28/08/06): Na, 75 meq/L; K, 28.3 mEq/L; creatinine: 50.4 mg/dL.

The child was started on sodium nitroprusside infusion and oral amlodepine. The intracranial pressure (ICP) was 70–80 mm Hg. Measures for raised ICP were instituted. A neurosurgery consultation was obtained and the hematoma was evacuated. Thereafter, he was shifted to intensive care unit. Post-evacuation, the ICP dropped to 30–35 mm Hg, BP came down and nitroprusside drip was discontinued. Later, the BP continued to drop to <5th centile requiring fluid boluses, dopamine and adrenaline infusions were started. Twelve hours postevacuation, ICP began to rise again and reached up to 180 mm Hg. A mannitol bolus was given and a repeat computerized tomography (CT) was done. Anti-edema measures (dexamethasone, lasix, mannitol and hyperventilation) were continued but raised ICP persisted. He had fever in the postoperative period. Ceftriaxone + sulbactum and cloxacillin were started empirically. He also developed hyperglycemia with polyuria, which was treated with insulin infusion and vasopressin. Despite all the measures, the cerebral perfusion pressure continued to remain low. The neurological status and shock progressively deteriorated and he expired on 31.08.06.

## Unit Diagnosis

Malignant hypertension? Renovascular

Intracranial bleed

## Dr. Munni Ray

This 12-year-old healthy adolescent male presented with sudden onset seizures, altered sensorium and vomiting without a preceding history of trauma or any bleeding manifestations. On examination he had severe hypertension, and the BP in the lower limbs was less that than in the upper limbs. He was comatose, had right sided hemiparesis, papillary edema with fundal bleed and there was raised ICP. Preterminally, renal dysfunction, hypernatremia and hyperglycemia developed. Calcium, phosphorus and alkaline phosphatase were within normal limits. The child had raised hemoglobin with normal coagulation profile and platelet counts. The urinanalysis was unremarkable. Radiological investigations showed intracranial bleed in the left basal ganglion region along with hydrocephalus, and ultrasound of the abdomen revealed a small left kidney with nephrolithiasis.

Hemorrhagic stroke presents similarly in adults and older children with features of raised ICP in the form of headache, vomiting and irritability, which rapidly progress to neurological deterioration and coma with seizures or hemiparesis. Presence of seizures goes more in the favor of hemorrhagic stroke than the ischemic stroke. Spontaneous intracranial bleeds are less common in children and can either be subdural, subarachnoid or intra-parenchymal.

Let us examine the risk factors of the hemorrhagic strokes in children. In sharp contrast to adults where systemic arterial hypertension is the most common cause of intracranial hemorrhage, in children more than one risk factor may be present. The commonest causes are arteriovenous malformation and aneurysms, followed by hematological problems and brain tumors. Here we do not have the radiology so we cannot comment on brain tumors. Hematological abnormalities were ruled out by investigations. The bleed was in anterior circulation, while aneurysmal bleed was more commonly subarachnoid in location. There are some genetic disorders which predispose to aneurysms like fibromuscular dysplasia, autosomal dominant polycystic kidney disease, but in this case, we have no such features. Aneurysmal bleed seems unlikely. Other causes like vasculitis can also present with intracranial hemorrhage.

There is no doubt that this child had significant hypertension. This is supported by the presence of neuro-retinopathy (fundal hemorrhages and retinal edema). Intracranial bleeding due to hypertension is typically due to the involvement of perforator vessels of the lenticulo-striate branches and is noted in the basal ganglion region as was seen here. This supports the possibility of hypertensive rather than aneurysmal bleed. Though hypertension is the commonest cause of hypertension in children in the adolescent age group, in view of very high BP, I would think more of a secondary hypertension, either renoparenchymal or renovascular, or due to vasculitis. One must also consider endocrine tumors like pheochromocytoma or Cushing’s disease as causes of childhood malignant hypertension.

The pediatric malignant hypertension series from India shows that the common causes are renoparenchymal diseases followed by renovascular hypertension. In the latter, aortoarteritis is the most important cause. Essential hypertension is rare. In this child, we cannot rule out renoparenchymal disease in view of unilateral small kidney; however, he had normal renal function tests at presentation. His urine examination was also normal. So it is very difficult to put renoparenchymal disease per se as the cause for malignant hypertension. But asymmetric kidney could be due to several reasons like pyelonephritis, chronic obstruction and unilateral vesico-ureteric reflux (VUR). VUR is one of the commonest causes of asymmetric kidneys in children. Congenital hypoplastic or dysplastic kidneys can also cause small kidneys. The small left kidney also showed nephrolithiasis. It is difficult to say what exactly was the cause of this calculus but stones that are typically present in the lower pole could be because of any genitourinary abnormality which causes stasis and persistent infection.

I think renovascular hypertension is a very strong possibility in this child. The differential diagnosis includes fibromuscular dysplasia, a non-inflammatory non-atherosclerotic disease. Mostly it involves the renal and carotid arteries. When it involves primarily the renal arteries, it leads to renovascular hypertension, and involvement of the cervico-cranial system can cause dissection as well as intracranial aneurysms. Fibromuscular dysplasia has to be ruled out from other genetic disorders like neurofibromatosis or Ehler Danlos syndrome but here we do not have any clinical manifestations for these possibilities. The lower limb BP was lower than that in the upper limb, so Takayasu’s arteritis should always be kept in mind. Though it is more common in females, it can occur in males also. Hypertension is a significant finding in such cases. Asymmetry of pulses is seen in only about 60% of the cases so its absence does not rule it out. Abdominal aorta involvement and renal artery involvement have been there in almost three-fourths of the cases. Children have almost similar presentation as adults or in some series they present earlier with less severe disease. Takayasu’s arteritis can be associated with some form of intracranial aneurysms which can cause intraparenchymal bleed. Finally, coarctation of aorta is much more common in the neonates and younger children. In teenage population, it is reported with neurofibromatosis type. The significant finding of pulse difference between the upper limb and lower limb is in favor of this, but cerebral hemorrhages described in neonates have not been seen in the adolescent population.

Another interesting finding was that this child had a hemoglobin level of 16.9 g/dL though later it became 14 g/dL. So I shall try to look into the causes based on which this polycythemia with hypertension could be explained. Cystic kidney diseases, renal artery stenosis or catecholamine secreting tumors are known to present with polycythemia and hypertension. There are case reports where paragangliomas are found to compress the renal arteries on one side leading to unilateral ischemic kidney with malignant hypertension. So, if we get to see anything like this in autopsy, we should not be surprised. The last thing is Von-Hippel Lindau syndrome where both the kidneys and the brain can be involved along with pheochromocytomas. Here also polycythemia has been described. These children generally have cerebellar hemangioblastomas rather than arterial circulation bleed. Certain vasculitidies like polyaretritis nodosa, Henoch Schonlein Purpura and systemic lupus erythematosus can present with malignant hypertension.

Preterminally, this child had a significant polyuria which could be explained by the hyperglycemia. The significant hypernatremia and polyuria could also be due to diabetes insipidus or diuretics which he got for raised ICP management. Once there is hypertensive crisis, a vicious cycle is set up leading to disturbances in autoregulation, which in turn can cause significant lowering of the cerebral perfusion pressure, gross cerebral edema leading on to ischemia and infarction and ultimately brain death.

## Dr. Ray's diagnosis

- Hemorrhagic stroke secondary to malignant hypertension.
- Renovascular hypertension either due to aortoarteritis or fibromuscular dysplasia.
- Unilateral small kidney with nephrolithiasis.
- Cause of death: refractory raised ICP.

**Dr. Sakhuja:** If I get to see patient like this, I would keep unilateral renoparenchymal disease as an obvious first cause for secondary malignant hypertension and everything else like the possibility of intimal fibroplasia, aortoarteritis or coarctation will all come after that possibility.

**Prof. S.N. Mathuria, Head, Department of Neurosurgery:** Definitely the cause of bleed is hypertension as it is the most important cause of a basal ganglia bleed, both in adults and in children. Aneurysms are uncommon in children but these are not rare or exceptional. At the same time, the aneurysm usually produces subarachnoid hemorrhage with or without intracerebral hemorrhage. If there is an aneurysm located in the M1 segment of the middle cerebral artery which is pointing medially or superiorly, it can produce a basal ganglia bleed. Also, if there is an aneurysm which is directed posteriorly and located at ICA bifurcation, it can also produce a basal ganglia bleed and the ICA bifurcation is the commonest sight to be seen in children. It is far more likely in children than in adults. Arteriovenous malformation (AVM) produces intracerebral hemorrhage more as compared to aneurysms and can be considered as an alternative possibility. Lastly, when the patient was referred to us, he was already decompensated (M2 with dilated pupils). Within 4 hours, the hematoma was evacuated but since the patient was already decompensated, he did not improve and he did not give us time to investigate further to find out the intracranial pathology.

**Dr. Sanjay Jain, Professor, Internal Medicine:** To differentiate between renal parenchymal or vascular disease, I always go by the kidney size. If the opposite kidney size is normal, I feel this is an acquired disorder. If the BP in the lower limb is less than the upper limb, naturally it would mean that abdominal aorta is involved. A small kidney and lower BP in lower limbs, suggest involvement of abdominal aorta and renal artery and the only thing that comes into my mind is Takayasu's arteritis.

**Dr. Vivek. Jha, Additional Professor of Nephrology:** According to the ultrasound report, the small kidney had a stone in it, which would strongly argue against the possibility of that kidney primarily being abnormal due to renovascular disease. One has to think of VUR having caused unilateral atrophy of the kidney. It is known that the reflux can be associated with malignant hypertension because of the distortion of the anatomy of renal vessels on that side. It can also cause infections giving rise to stone. So this possibility will be higher than the possibility of renovascular hypertension. Rest of the events that followed were obviously due to accelerated hypertension.

**Dr. Rajesh Vijayvergiya, Assistant Professor of Cardiology:** I just want to enquire whether this child was given any ACE inhibitors to control the BP as the deteriorating renal functions after ACE inhibitors can give a clue to bilateral renal artery stenosis.

**Dr. Ray:** This child received only nitroprusside and amlodepine.

**Dr. Sakhuja:** This patient developed raised ICP again after the evacuation of hematoma and we found cerebral edema in the last CT scan, I do not know why that developed. There may have been component of sepsis also since this patient became febrile preterminally. Another odd feature is the development of hyperglycemia in the last 2–3 days of his hospital stay.

**Dr. Mathuria:** Actually once there is a hemorrhagic stroke then the surrounding brain shows the edema and we only take out the hematoma. Particularly in the basal ganglia region, we cannot provide much decompression; so the edema will persist.

**Dr. Pratibha Singhi, Professor of Pediatrics:** Hyperglycemia can occur with sepsis and severe stress; it is not uncommon in children, who even require insulin and drugs. One could always think of unusual things like pheochromocytoma which can cause malignant hypertension, hyperglycemia, polycythemia and a sudden elevation after a sympathetic stress and they are also associated with Von-Hippel Lindau disease which may be associated with aneurysm or AVM in the brain but certainly small kidney cannot be explained.

## Dr. Amanjeet

In this 12-year-old male, a complete autopsy was performed. The pleural, peritoneal and pericardial cavities were within normal limits.

**Figure 1:**
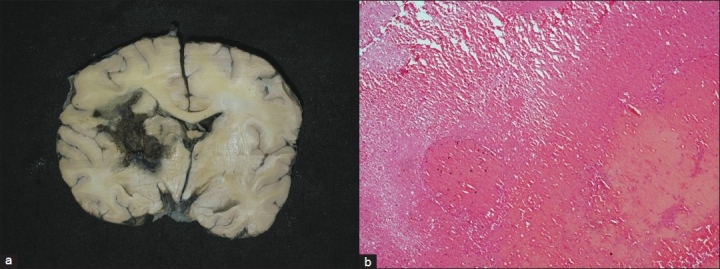
a) Coronal slice of brain showing left sided haemorrhage in the region of putamen, extending to central white matter, thalamus and posterior limb of internal capsule; b) Photomicrograph showing fresh ganglionic haemorrhage and oedema in adjacent brain parenchyma (H and E, ×250)

The brain [Figure 1] weighed 1000 g. There was evidence of incision mark on cranial cavity from the left frontoparietal to occipitoparietal region. The brain showed marked cerebral edema. There was evidence of extradural hemorrhage on the left occipitoparietal region along with the suture mark and this can be related to procedure done on this child. There was evidence of bilateral uncal herniation. The circle of Willis did not show any occlusion or aneurysmal dilatation of the vessels. The coronal slice of brain showed hemorrhage in the putamen, extending into thalamus, posterior limb of internal capsule as well as the subcortical white matter. No extension into the ventricle cavity was seen. The adjacent brain parenchyma showed softening and edema, possibly because of ischemic changes in the surrounding areas of hemorrhages. Microscopic section showed intraparenchymal fresh hemorrhages with ill-defined margins. The surrounding parenchyma showed marked edema with perineuronal vacuolization. Parenchymal blood vessels were stiff, there was thickening of vessels wall with sclerosis and they were opened up in comparison to the collapsed blood vessels seen in the normal brain parenchyma. There was fibrinoid necrosis of the vessel wall with surrounding edema and vacuolization. However, there was no inflammatory infiltrate to suggest vasculitis. In other areas away from the main site of hemorrhage, there were microscopic perivascular hemorrhages.

**Figure 2:**
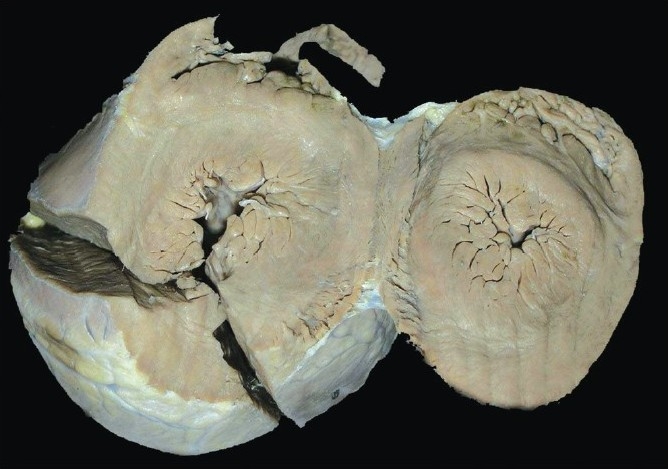
Apical slice of heart showing concentric left ventricular hypertroph

The heart weighed 140 gm and was globular in shape. There was prominence of the left border and a few epicardial hemorrhages. The left ventricular wall thickness was 1.5 cm. The right ventricular wall thickness was 0.4 cm. Apical slice of the heart showed marked concentric left ventricular hypertrophy [Figure 2] and on microscopic examination here was mild anisonucleosis of myocardial fibers; however, there was no evidence of interstitial fibrosis or myofibral disarray. There were subendocardial hemorrhages and grade II atherosclerosis in the aorta.

**Figure 3:**
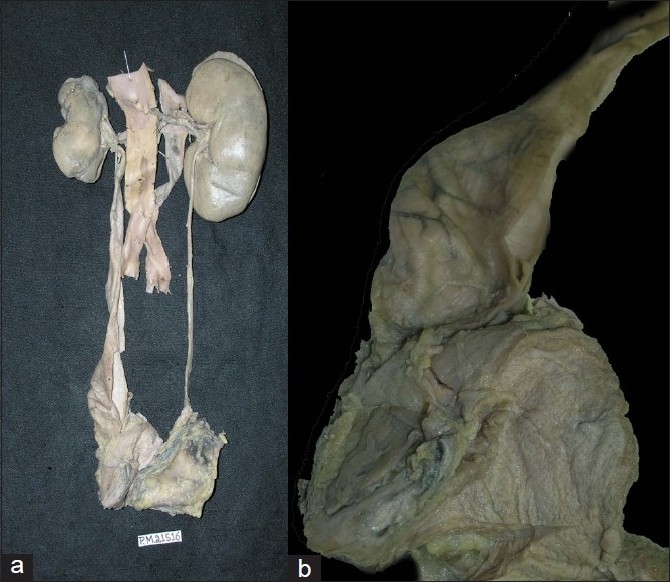
a) Gross photograph showing small and shrunken left kidney with marked dilatation of ureter through out its length and normal right kidney and ureter; b) Left ureter showing maximum dilatation at lower end

**Figure 4:**
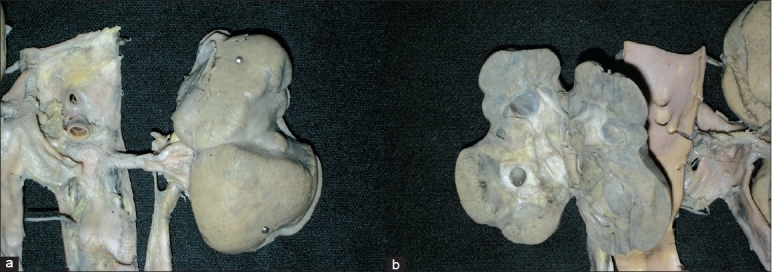
a) Gross photograph of left kidney with deep transverse cortical scars; b) Cut surface showing dilatation of pelvicalyceal system and thinned out cortex in scar area

**Figure 5:**
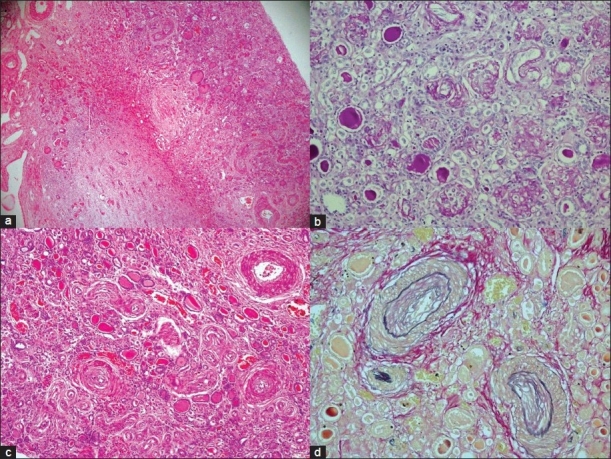
a) Photomicrograph showing scar area of left kidney (H and E, ×250); b) Scar shows globally sclerosed glomeruli, tubular atrophy with thyroidation and endocrination (H and E, ×440); c) Extensive fibrointimal proliferation and narrowing of lumina involving arterioles (PAS, ×440), small terminal interlobular arteries; d) Vascular changes highlighted on EVG stain (×440).

**Figure 6:**
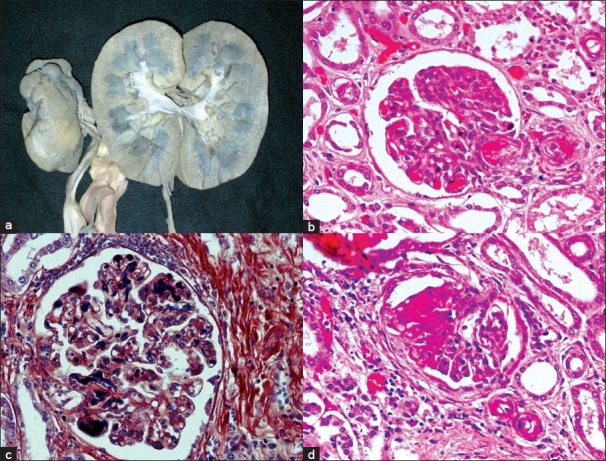
a) Gross photograph of cut surface of right kidney showing medullary congestion; b) Afferent arteriole and glomerular capillaries showing features of thrombotic microangiopathy (H and E, ×440); c) PTAH stain highlighting fibrin thrombi in glomerular capillaries (×440); d) Segmental sclerosis of glomerular tuft (H and E, ×440)

The left kidney was small and scarred, while the right kidney was normal in size [Figure 3a]. The left ureter showed marked dilatation throughout its length [Figure 3b], the right ureter was normal. The closer view of the left kidney showed deep coarse scars on the upper pole as well as in the lower pole [Figure 4], there was a deep transverse groove in the middle portion of the left kidney. The cut section showed dilatation of pelvicalyceal system (PCS), and marked thinning of the cortex adjacent to the dilated pelvicalyceal system; however, no stone was recovered from the lower pole. The microscopic examination of PCS showed mild inflammation and fibrosis. Vessels in the scarred area showed fibrointimal proliferation and concentric fibrosis giving them onion skin appearance [Figure 5]. In addition, there was marked atrophy of the tubules. They were lined by flattened epithelial cells and there was presence of hyaline casts. The interlobular arteries showed marked fibrointimal proliferation and there was interstitial fibrosis. Elastic von Giessen stain showed fibrointimal proliferation and duplication of the internal elastic lamina [Figure 6]. Some glomeruli were globally sclerosed and in few areas the tubules were closely packed to each other without significant interstitial fibrosis indicating endocrinization which reflects the ischemic change. The junction of the scar and the normal parenchyma was sharply demarcated. Adjacent parenchyma showed fibrin thrombi within the capillary lumina extending into afferent arterioles with fibrinoid necrosis of the wall of arterioles. In addition to thrombotic microangiopathy, the vessels in the preserved renal parenchyma showed fibrointimal proliferation and concentric layering of the fibrous cells in arterioles. In some areas, tubules showed calcification; however, no oxalate crystals were seen either on light microscopy or under polarizing light.

**Figure 7:**
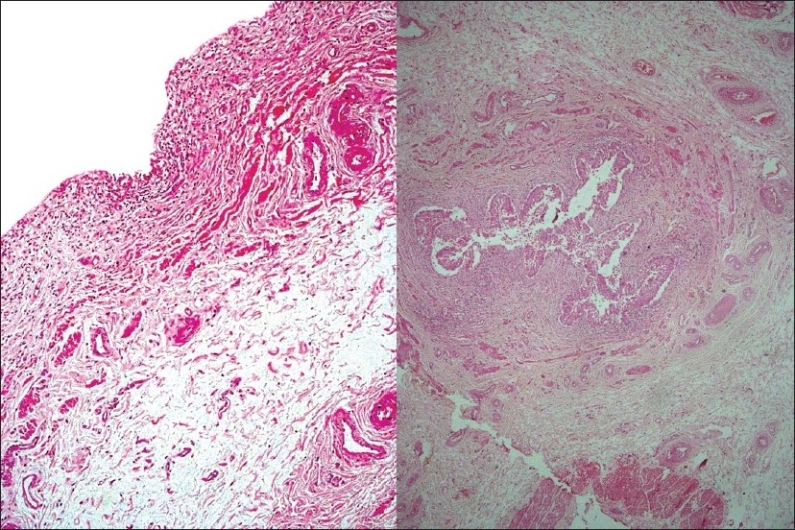
Photomicrographs showing flattened lining in the dilated ureter with inflammatory infiltrate and thinning of the muscularis propria (H and E, ×120)

The left ureter was markedly dilated throughout its length, more so in the lower 5–6 cm. However, the ostia and opening of the ureter were patent. The lining was flattened in the dilated ureter and there was mild inflammatory infiltrate and thinning of the muscularis propria [Figure 7]; however, the intravesical part of the ureter showed patent lumina, ruling out ureteric stenosis at the vesico-ureteric junction.

The opening of the left renal artery was patent, there was no atherosclerotic change or significant stenosis. On microscopic examination of left renal artery, the lumen was not significantly narrowed, the media of the vessel showed moth-eaten appearance, i.e. the pale areas alternating with the dark areas which represent the smooth muscles. On Masson's trichome stain, the media of the left renal artery was replaced by fibrosis; however, the right renal artery showed a normal media. The closer view showed fiber disarray of the smooth muscles of the media and fibrous replacement of the media indicating medial fibroplasia.

Right kidney was non-scarred, but appeared slightly swollen and showed tiny hemorrhagic areas. On cut section, the corticomedullary junction was distinct, there was marked medullary congestion and the pelvicalyceal system was normal. The right kidney also showed changes of thrombotic microangiopathy. Some of the glomeruli also showed secondary focal segmental glomerulosclerosis. The right renal artery was microscopically unremarkable, the media was normal, the internal elastic lumina as well as external elastic lamina were intact.

Both the lungs were heavy. Slicing showed hemorrhagic consolidation in both lungs. Microscopy showed marked intra-alveolar capillary congestion, evidence of aspiration, along with the spores and pseudohyphae of the candida. The pulmonary artery branches showed fibrin thrombi and pulmonary edema.

The adrenals showed focal hemorrhages. There was no tumor.

## Final Autopsy Diagnosis

- Unilateral reflux nephropathy with megaureter.
- Changes of malignant hypertension in the form of thrombotic microangiopathy, intracerebral hypertensive hemorrhages and left ventricular hypertrophy.
- Pulmonary thromboembolism, pulmonary edema, aspiration and oesophageal candidiasis.

**Dr. Sakhuja:** It appears that the hypertension was of considerable duration as evidenced by concentric left ventricular hypertrophy that we have seen and therefore was most probably secondary to the unilateral abnormality of the kidney leading to the malignant hypertension which in turn led to the intracerebral bleed and thrombotic microangiopathy.

This patient underwent ICP monitoring; which is something not very commonly done. Now the importance of this is whenever you have raised ICP, in order to maintain cerebral perfusion pressure you must ensure that the mean arterial pressure remains high and intracerebral bleed is the situation which is associated with the maximum increases in ICP. So, in order to maintain cerebral perfusion pressure, the BP should not be lowered rapidly, in fact it should be kept at the higher level. Only then, the cerebral perfusion pressure will remain adequate. But I would like the pediatricians or neurosurgeons to comment on the situations where they use ICP monitoring.

**Dr. Singhi:** Actually ICP monitoring is now being used mainly to maintain the cerebral perfusion pressure. In fact until recently, the management of raised ICP was targeted toward bringing down the ICP by various modalities including osmosis, hyperventilation, etc. Recent insights show that you have to target the cerebral perfusion pressure. So, in our department, studies have been carried out on ICP monitoring which has been found to be feasible even in children with CNS infections and in fact that study was published last year and subsequently we have compared the outcomes with ICP targeted therapy vs CPP targeted therapy and the trends favor CPP target therapy. So the ideal is to manage with the CPP targeted therapy.

